# Supplementary material for: Assessing Attitudes and Participation Regarding a Pilot COVID-19 Workplace Vaccination Program in Southern Germany Considering the Occupational Health Perspective—A Mixed Methods Study
Source: Vaccines (Basel). 2023 Jun 9;11(6):1082. doi: 10.3390/vaccines11061082 (PMC10304481; doi:10.3390/vaccines11061082)
Supplement: Supplementary file 1 [file vaccines-11-01082-s001.zip › vaccines-2415614-supplementary.pdf]

Vaccines

Article

Assessing Attitudes and Participation Regarding a Pilot COVID-19 Workplace Vaccination Program in Southern Germany Considering the Occupational Health Perspective—A Mixed Methods Study

Anke Wagner, Kamil Keles, Christine Preiser, Anna Neunhöffer, Jana Soeder, Juliane Schwille-Kiuntke, Monika A. Rieger and Esther Rind

Supplementary Materials

Content of Supplementary Materials

- **Table S1.** Interview guide for single interviews
- **Table S2.** Category system including main categories, subcategories, and definitions

Table S1. Interview guide for single interviews

Note: Translated to English by the authors for the purpose of publication. Original version was in German only.

Structure of the interviews	Content of the interviews
**1. Introduction**	• Welcome and thanks for participation<br>• Information about the study<br>• Information about the interview (aim of the interview, presumed duration, request to freely describe your impressions, no right or wrong answers)<br>• Brief repeated information on data protection
**2. Organization of the early COVID-19 workplace vaccination program**	Key questions<br>• How did you personally experience the start of the early COVID-19 workplace vaccination program?<br>• How did you organize and implement the vaccinations in your company?<br>• What was your personal role during the process of your company becoming part of the early COVID-19 workplace vaccination program?<br>• What support did you receive in planning and implementing vaccination as part of the early COVID-19 workplace vaccination program?<br>• How do you assess the cooperation with the Ministry of Social Affairs in the context of the early COVID-19 workplace vaccination program?<br>Follow-up questions<br>• What specific support did you receive?<br>• What kind of support do you wish you had received?<br>• What would have been helpful in retrospect?<br>• What other support did your company receive?
**3. Perceived response of employees to the early COVID-19 vaccination offer**	Key question<br>• How was the offer for vaccinations received by the employees in the early COVID-19 workplace vaccination program?<br>Follow-up question<br>• Which employees had a particular need for information?
**4. Identified advantages and disadvantages of COVID-19 vaccinations in companies**	Key questions<br>• What advantages can you see in occupational physicians contributing to the COVID-19 vaccination campaign in companies?<br>• Which disadvantages exist from your point of view?

Structure of the interviews	Content of the interviews
**5. General assessment of the early COVID-19 workplace vaccination program**	• What benefit do you think the early COVID-19 workplace vaccination program has for workplace vaccinations outside of this?
**6. Implications based on the early COVID-19 workplace vaccination program**	Key questions<br>• How has vaccinating against COVID-19 affected your everyday work?<br>• Which occupational health care topics do you think have become more important and urgent during the pandemic?<br>• Which work activities moved into the background?<br>• What do you personally take into account for the future for the companies where you provide occupational health care?<br>• What would you like to see in your future in general?
**7. Final question and conclusion**	• Are there any aspects that have not been mentioned but that you would like to address?<br>• Thank you for participating in the interview.

Table S2. Category system including main categories, subcategories and definitions

Note: Translated to English by the authors for the purpose of publication. Original version was in German only.

**Main categories**	**Subcategories**	**Definition**
Vaccination during the early COVID-19 workplace vaccination program	Experiencing the start of the early COVID-19 workplace vaccination program	All statements that refer to the start of the early COVID-19 workplace vaccination program and how the interviewees perceived it.
	Preparation of the COVID-19 vaccinations	All statements relating to the preparation of COVID-19 vaccination as part of the early COVID-19 workplace vaccination program, be it organizing facilities, scheduling appointments, information campaigns, handling the COVID-19 vaccines (transport and storage), and prioritization of persons to be vaccinated.
	Carrying out the COVID-19 vaccinations	All statements that refer to the implementation of COVID-19 vaccinations as part of the early COVID-19 workplace vaccination program and related activities that were carried out in advance and afterwards.
Support and cooperation during the early COVID-19 workplace vaccination program	Support and cooperation	All statements related to support and cooperation: internal support, external support, as well as cooperation with vaccination centers and Ministry of Social Affairs, Health and Integration.
	Need for support	All statements regarding possible support needs during the early COVID-19 workplace vaccination program.
Perceived response of employees to the early COVID-19 vaccination offer	Positive response	All statements referring to a positive response to COVID-19 vaccination in the context of the early COVID-19 workplace vaccination program.
	Negative response	All statements referring to a negative response to COVID-19 vaccination in the context of the early COVID-19 workplace vaccination program.
Change in everyday working life	Workload	All statements on workload during the early COVID-19 workplace vaccination program and the period afterwards.
	Occupational health issues	All statements on special occupational health issues that are suddenly more the

		focus of an occupational physician's activities.
	Occupational health and safety measures	All statements on special occupational health and safety measures implemented in the company.
Identified advantages and disadvantages of COVID-19 vaccinations in companies	Advantages	All statements dealing with the advantages of vaccination against COVID-19 through occupational physicians.
	Disadvantages	All statements dealing with the disadvantages of vaccination against COVID-19 through occupational physicians.
General assessment of the early COVID-19 workplace vaccination program	The early COVID-19 workplace vaccination program	All statements that refer to a general assessment of the early COVID-19 workplace vaccination program, both critical attitudes and positive attitudes, are included.
	Implications and wishes	All statements that include implications that have resulted from the COVID-19 vaccinations in workplaces or overriding wishes for the future.
